# The framework for systematic reviews on psychological risk factors for persistent somatic symptoms and related syndromes and disorders (PSY-PSS)

**DOI:** 10.3389/fpsyt.2023.1142484

**Published:** 2023-04-05

**Authors:** Paul Hüsing, Abigail Smakowski, Bernd Löwe, Maria Kleinstäuber, Anne Toussaint, Meike C. Shedden-Mora

**Affiliations:** ^1^Department of Psychosomatic Medicine and Psychotherapy, University Medical Center Hamburg-Eppendorf, Hamburg, Germany; ^2^Department of Psychology, Utah State University, Logan, UT, United States; ^3^Department of Psychology, Medical School Hamburg, Hamburg, Germany

**Keywords:** persistent somatic symptoms, somatic symptom disorder, bodily distress disorder, functional somatic syndromes, psychological risk factors

## Abstract

**Introduction:**

Numerous psychological factors are believed to play a pivotal role in the development and maintenance of persistent somatic symptoms (PSS) in all fields of medicine. However, very few of these factors have been empirically investigated in relation to PSS. The aim of this study is firstly to propose a framework and define search terms for systematic reviews on the empirical evidence and diagnostic value of psychological risk factors for PSS and PSS-related outcomes (PSY-PSS). Secondly, the application of the framework is illustrated using the example of a systematic review on the relevance of psychological factors in somatic symptom disorders (SSD; DSM-5) and bodily distress disorders (BDD; ICD-11).

**Methods:**

Following a narrative review approach, two comprehensive lists of search terms to identify studies in (1) relevant patient groups with PSS and (2) relevant psychological factors were generated by reviewing the current literature and employing an iterative process of internal revision and external expert feedback.

**Results:**

We identified 83 relevant symptoms, syndromes and disorders for which we defined a total of 322 search terms (list 1). We further comprised 120 psychological factors into 42 subcategories and 7 main categories (list 2). The introduced lists can be combined to conduct systematic reviews on one or more specific psychological factors in combination with any symptom, syndrome or disorder of interest. A protocol of the application of this framework in a systematic review and meta-analysis on psychological etiological factors of SSD and BDD is presented following the PRISMA guidelines.

**Discussion:**

This framework will help to gather systematic evidence on psychological factors in order to improve the understanding of the etiology of PSS, to refine future diagnostic conceptualizations of PPS, and to develop optimized mechanism-based interventions for individuals with PPS and related syndromes and disorders.

## Introduction

Persistent somatic symptoms (PSS) are a common phenomenon. Up to 80% of the general population report one or more somatic symptoms over the course of the past 4 weeks (1–3). While in most cases symptoms are remittent, they persist in approximately one fourth of all patients. These symptoms often impair patients’ lives even years after initial appearance, regardless of their origin or underlying physiopathology (4, 5). PSS are common in almost all medical specialties and many times remain “medically unexplained.” Even though the term “medically unexplained symptoms” (MUS) has been used in clinical practice and research for many years, the concept was considered problematic since the reliability of assessing whether or not there is a pathophysiological explanation for a certain symptom is notoriously poor. Furthermore, the concept reinforced a mind–body-dualism, the fact that a symptom cannot be “medically explained” does not imply that it must be part of a psychiatric disorder (6). Regardless of their etiology, PSS pose a challenge in medicine regarding diagnostic accuracy, early detection and appropriate treatment. Repeated medical examinations and invasive treatments are common, despite being time-consuming and costly (7).

Current evidence regarding the etiology of PSS defines biomedical and psychosocial predisposing, triggering, and maintaining/aggravating factors (8–10). Thus, a thorough diagnostic process based on a biopsychosocial perspective is essential for successful treatment (11, 12). Most etiological models of somatoform and functional disorders include psychological factors involving cognitive-perceptual mechanisms such as amplified perception of bodily sensations or selective attention processes, affective factors such as illness anxiety or emotion regulation deficits, and behavioral mechanisms such as avoidance (13–16). The ICD-10 and DSM-IV classification of somatoform disorders did, however, not include any psychological criteria, and diagnosis was thus merely based on the presence of symptoms in the absence of a medical explanation (17, 18). Similarly, the diagnostic conceptualizations of the most common functional syndromes, i.e., irritable bowel syndrome (19), fibromyalgia (20) and chronic fatigue syndrome (21), do not contain any psychological factors at all.

In the revised diagnostic concepts of somatic symptom and related disorders in DSM-5 (22), and bodily distress disorders in ICD-11 (23), psychological criteria are now included. Somatic Symptom Disorder (SSD) is defined by one or more persistent somatic symptom(s) that are distressing or result in significant disruption of daily life. Symptoms may or may not be medically explained (24). The new diagnostic concepts now require that the somatic complaints are accompanied by excessive and disproportionate health-related thoughts, feelings, and behaviors.

The introduction of these psychological criteria was supported by evidence suggesting it is not the somatic symptoms *per se* that result in suffering and increased health care needs, but rather how patients interpret and act upon the symptoms (25, 26). In fact, psychological features such as health anxiety and catastrophizing are significant determinants of disability, health care utilization, and predict disease course and treatment outcome (27–29). Experts in the field thus welcomed the inclusion of psychological criteria to the diagnostic classification of PSS in DSM-5 and ICD-11 (24, 25, 30). A recently published scoping review on the empirical evidence of somatic symptom disorder summarized the generally good reliability, validity and clinical utility of the DSM-5 criteria (24). However, it was also pointed out that ‘the greatest need for improvement of the SSD diagnostic criteria appears to be measurable and more precise diagnostic B-criteria’ (24), i.e., the psychological ‘positive’ criteria of the diagnosis. Further, the choice of the psychological criteria has been criticized (31), and the relevance of the clinical context and subjective interpretation of these criteria was highlighted (32, 33).

There is empirical evidence for a long list of further psychological variables relevant to PSS and related conditions, that have not been included in the DSM-5 and ICD-11 diagnostic criteria. The following paragraph will provide several relevant examples. Regarding behavioral factors, fear avoidance behavior is among the best predictors for the transition of acute to chronic pain (34). For patients with multiple somatic symptoms, avoiding situations that challenge the body is one of the most powerful variables distinguishing highly disabled patients from those with low health care needs, even if both groups report a similar number of somatic symptoms (35). Behavioral avoidance is furthermore related to physical inactivity and subsequent deconditioning (36). As cognitive determinants of PSS, ruminations about physical complaints, self-concept of bodily weakness, and subjective low symptom tolerance have been suggested (35). Affective symptoms and traits such as negative affectivity, desperation, hope-and helplessness, anger related to somatic symptoms, as well as deficits in emotion regulation, in particular alexithymia, are further relevant factors in the maintenance of somatic symptoms (37). Empirical data also support the importance of selective attention processes, or amplified perception of bodily sensations in the sense of somatosensory amplification in the perseverance of symptoms (13, 38–40). Further potentially aggravating factors of PSS arise from unsatisfying interactions between patients and the health care system, negative illness perceptions, and treatment experiences (41), which oftentimes result in unnecessary and potentially harmful extensive utilization of health care (42).

Since most studies investigate singular psychological factors and focus on singular PSS-related diagnostic conditions, the data for most of these psychological criteria are not sufficiently robust. As the evidence has not been systematically reviewed, there is no consensus on their potential justification in the diagnostic classifications of PSS and related syndromes and disorders. In summary, one of the greatest needs for the improvement of the diagnostic classification for PSS across medical fields is to identify the evidence on those psychological factors. This evidence should either show that a specific psychological factor is able to discriminate patients suffering from PSS and related conditions from healthy or clinical controls, or be significantly associated to PSS-relevant clinical outcomes such as symptom severity, functional impairment, quality of life or health care utilization. In the sense of a risk factor, a psychological factor should be predictive for these outcomes or for the development or maintenance of the symptoms and conditions themselves. This framework provides the tools to systematically collect this evidence.

### Aims and objectives

It is our overall aim to systematically review the scientific evidence of pre-defined psychological variables in relevance to PSS, somatic symptom disorder, bodily distress disorder, functional syndromes and related disorders. This will require a number of individual systematic reviews and meta-analyses addressing different psychological variables in combination with different PSS and related syndromes and disorders. Results will ultimately provide an improved etiological understanding of PSS, and consequently be a starting point for an improvement of diagnostic conceptualizations, diagnostic validity and the development of mechanism-based interventions.

Within this paper, we pursue two aims. First, we present a framework for systematic reviews in order to evaluate the empirical evidence and diagnostic value of psychological correlates and risk factors for PSS and related syndromes and disorders (PSY-PSS). To this end, we developed two lists of relevant search terms in this research area, i.e., an extensive list of all relevant search terms for PSS and related syndromes and disorders and a comprehensive list with potentially relevant psychological factors. Both lists can be combined to conduct systematic reviews on specific psychological factors and specific PSS and related syndromes or disorders and thus provide researchers with a common search matrix as starting point.

Second, based on our PSY-PSS framework, we present the study protocol of a first systematic review on the identification of psychological associates and risk factors for the most recent diagnostic concepts of PSS, i.e., somatic symptom disorder (SSD; according to DSM-5) and bodily distress disorder (BDD; according to ICD-11). In the future, we aim to follow up with further systematic reviews on the relevance of certain psychological factors such as negative affectivity or avoidance behavior in other PSS-related syndromes and disorders, such as irritable bowel syndrome, chronic fatigue syndrome, or fibromyalgia.

## Methods and results

In a first step, we identified the diagnostic terms used for PSS and related syndromes and disorders throughout various fields of medicine. In a second step, we narratively reviewed the current literature to identify potentially relevant psychological associates and risk factors for PSS and related syndromes and disorders.

### Lists of PSS and related syndromes and disorders and identified psychological factors

#### PSS and related syndromes and disorders

Our goal was to create a comprehensive list of diagnostic terms used for PSS and related syndromes and disorders in research and clinical practice. Thereby, we aimed to cover all diagnostic classifications that are based on the presence of persistent somatic symptoms, regardless of their etiology. First, we included all psychiatric and general diagnostic concepts, i.e., somatoform disorders (DSM-IV and ICD-10), somatic symptom and related disorders (DSM-5), bodily distress disorders (ICD-11), and so-called “medically unexplained symptoms.” Second, we included functional somatic syndromes from all medical specialties (e.g., irritable bowel syndrome, fibromyalgia, chronic fatigue syndrome). Third, in addition to clinically established diagnoses, we also included syndromes from specific health care systems, such as bodily distress syndrome (43), wind turbine syndrome, or chronic whiplash syndrome, and Long Covid.

Our list is based on a previous list created for a systematic review on early interventions for PSS (44), which in turn was based on a list published earlier by Henningsen et al. (45). We reviewed this list with a team of experts (MSM, AT, AS, PH), added missing terms, and rearranged the terms into diagnostic subgroups based on symptom domains, i.e., by conditions and medical specialties (see Table 1). As the terminology for PSS and related syndromes and disorders differs substantially across countries and medical specialties, we tried to provide a comprehensive list of all clinically and scientifically terms used in Western countries (6, 46). The full list includes 83 conditions with 322 terms (Supplementary material Table 1).

**Table 1:** List of medical specialties and conditions (*n* = 83) included in the search list on PSS and related syndromes and disorders.

Specialty	Condition
General (non-specific) terms	Functional
General practitioner	Subjective symptoms, medically unexplained symptoms
Psychiatry/psychosomatic medicine	Dissociative disorders, somatoform disorder, somatization disorder, pain disorder, conversion disorder, somatic symptom disorder, bodily distress disorder, culture-bound syndrome
Allergology	Food intolerance, multiple chemical sensitivity, sick building syndrome, Persian Gulf syndrome, amalgam hypersensitivity, Implant intolerance, prothesis intolerance, aerotoxic syndrome, wind turbine syndrome, electromagnetic hypersensitivity
Anesthesiology	Idiopathic pain, chronic postoperative pain
Cardiology	Atypical chest pain, palpitations with normal investigations, syndrome X
Dermatology	Psychogenic skin disease
Endocrinology	Hypoglycemia
Gastroenterology	Functional gastrointestinal disorders, disorders of the gut-brain-interaction, functional bowel syndrome, nonulcer dyspepsia, functional abdominal pain, functional colon disease, functional disorders of swallowing, globus syndrome
Gynecology and urology	Premenstrual syndrome, functional urologic disorders, paruresis, dysfunctional voiding, idiopathic overactive bladder, interstitial cystitis, urethral syndrome, chronic pelvic pain syndrome, pelvic arthropathy
Infectiology	Chronic lyme disease, candida hypersensitivity, chronic rhinopharyngitis
Neurology	Functional seizures, functional voice disorder, functional motor disorder, functional eye movement disorder, functional facial movement disorder, functional tongue movement disorder, functional sensory symptoms, functional visual symptoms, functional speech disorder, functional memory disorder, functional cognitive disorder, functional dizziness, functional stroke, tension headache, atypical face pain, central sensitivity syndrome, post-concussion syndrome, chronic fatigue syndrome, myalgic encephalomyelitis, neurasthenia, post-viral fatigue syndrome
Oral medicine/otorhinolaryngology	Temporomandibular joint disorder, atypical odontalgia, psychogenic gagging, burning mouth, bruxism
Orthopedics	Repetitive strain injury, chronic whiplash syndrome
Respiratory medicine	Hyperventilation syndrome
Rheumatology	Fibromyalgia, chronic low back pain, chronic pain, persistent pain, chronic intractable benign pain syndrome

#### Psychological risk factors

**Figure 1:**
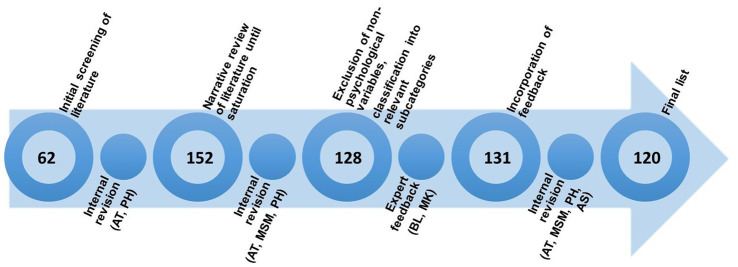
Process of developing the list of psychological factors. The numbers within the circles represent the number of terms included at the various stages.

Regarding the psychological risk factors, we employed a narrative review approach by searching the scientific literature, overview articles and books on the etiology, diagnosis and treatment of PSS, somatoform disorders and functional syndromes. A more systematic approach to derive at the psychological factors would have been desirable, but was not possible due to the heterogeneity of concepts and empirical evidence. The identified terms were reviewed and amended by experts and synthesized into categories in an iterative process. An overview of this process can be found in Figure 1 and will be described in further detail below.

**Figure 2:**
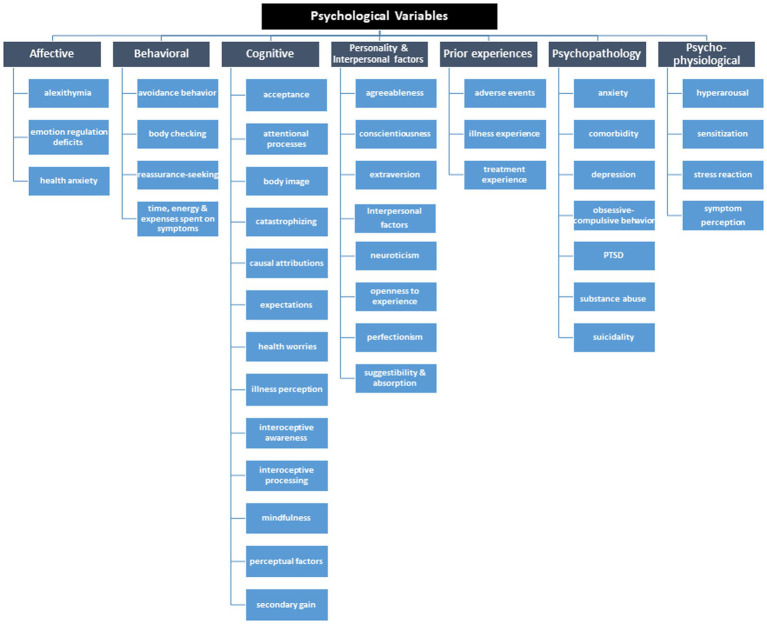
Tree diagram of relevant psychological variables potentially associated with PSS and related syndromes and disorders.

First, we screened current literature and overview articles to summarize known and assumed psychological factors with an influence on the development and maintenance of PSS, somatoform disorders, somatic symptom disorders, medically unexplained symptoms, and functional syndromes (10, 26, 30, 40, 47, 48). We used this list of *n* = 62 identified psychological variables as a starting point to review further literature until we reached saturation (total number of variables: *n* = 152). A group of three experts in the field of PSS (AT, MSM, PH) reviewed this list and classified each term within a subcategory. We excluded non-psychological factors, i.e., sociodemographic factors such as age or gender, or social factors such as social support, resulting in *n* = 128 variables. In a next step, we asked two independent international experts (BL and MK) to rate 128 psychological factors based on their relevance for PSS and related syndromes and disorders (both from a research and clinical perspective) and fit to subcategory, and asked for further potentially missing variables. Incorporation of expert feedback led to a total of *n* = 131 variables. We organized the terms into main and subcategories. After a last internal review and discussion (AT, MSM, PH, AS), we agreed on a list of 120 psychological variables in 42 subcategories and 7 main categories (see Figure 2; full list in Supplementary material Table 2).

#### Resulting research matrix for investigating psychological factors in PSS and related syndromes and disorders

We propose the application of the two lists in the sense of a framework that enables researchers to generate comparable evidence on psychological factors relevant to PSS and related syndromes and disorders. In order to foster common knowledge, both lists shall be freely available to other research groups, and are shared within the Open Science Framework (49, 50). The search strategy was developed to use each psychological variable (list 2) as a keyword in combination with the terms of the disorder-specific synonyms (list 1) using the logical operator AND. Psychological terms should be combined using the logical operator OR. Table 2 displays the idea of combining both lists in order to improve the evidence on psychological correlates and risk factors of PSS and related syndromes and disorders.

**Table 2:** Matrix for systematic searches to improve the evidence on psychological factors relevant to PSS and related syndromes and disorders.

Psychological factors (examples)		PSS terms (examples)			
		Somatic symptom disorder	Irritable bowel syndrome	Fibromyalgia	…
Behavioral	Avoidance behavior	Avoidance behavior in SSD	**…**		
Affective	Alexithymia	**…**			
Cognitive	Catastrophizing				
…	**…**				

### Application example of the PSY-PSS framework: Study protocol for a systematic review on the evidence of psychological concepts with prognostic validity in somatic symptom disorder and bodily distress disorder

Based on the generated framework, we aim to conduct a systematic review on the empirical evidence of psychological correlates and risk factors in the diagnoses according to the current classification systems DSM-5 (somatic symptom disorder; SSD) and ICD-11 (bodily distress disorder; BDD). The method and reporting of the review will be conducted in accordance to the guidelines outlined in the PRISMA statement (51).

#### Search strategy for the identification of relevant studies

Search terms will comprise of SSD or BDD, respectively, in combination with all terms from our list of psychological risk factors (Supplementary material Tables 1, 2). The literature search will include records from the following databases: PubMed (NCBI), PsycINFO (Ovid), Web of Science (Clarivate Analytics), as well as the Cochrane Database of Systematic Reviews.

We will review titles and abstracts of all identified studies. Due to the scope of the study and to ensure that we exclude outdated data, we will only include studies that were published after initial release of the DSM-5 criteria, i.e., from 2009 and onwards. We will only include studies published in English or German language in peer-reviewed journals.

#### Criteria for inclusion and exclusion of studies in the review

In order to be included in the planned review, identified studies must contain original data or be systematic reviews. Included studies or reviews must be based on patient populations diagnosed with SSD or BDD. Thereby, diagnoses should be given through either diagnostic interviews, clinical judgment, or validated screening measures. They should provide observational cross-sectional or longitudinal data on the association of psychological variables on diagnosis or disorder-relevant outcomes, i.e., symptom severity, impairment or quality of life. Psychological factors should be measured quantitatively. We will exclude studies that refer to former classifications of PSS, i.e., somatoform disorders, or use unclear methodology to provide diagnoses of SSD or BDD.

#### Quality assessment

The quality of all studies included in the analysis will be evaluated using the Effective Public Health Practice Project Quality Assessment Tool (52, 53). Two reviewers will evaluate the studies with regard to selection bias, study design, confounders, blinding, data collection methods, and withdrawals and dropouts. Any disagreement among the reviewers will be resolved with discussion and input from a third reviewer, if necessary.

#### Study coding and extraction of data

Two reviewers will review the titles and abstracts of the identified studies independently and ensure duplicates are removed. Decisions for or against inclusion will be based on the standardized inclusion/exclusion criteria. If not excluded in the initial round, full manuscripts will be retrieved, or in case where information in the title and abstract is not sufficient to exclude the study. In the second round, studies will be re-reviewed, data extracted using the automation software package Rayyan (54) and results will be compared and discussed by both reviewers. In case of disagreement, a third independent reviewer will be asked for mediation. The following data will be extracted from each included study (see Table 2):

1. Study characteristics: design, setting, primary aim of the study etc.
2. Diagnosis investigated in the study and operationalization of diagnostic criteria (SSD or BDD)
3. Psychological factors included in the study and operationalization of constructs (employed self-report instruments)
4. Disorder-relevant outcomes and their assessment along with effect sizes (if reported)

#### Descriptions of outcomes

In order to determine the relevance of the psychological variables as associates or risk factors for PSS in SSD and/or BDD, diagnostic status, somatic symptom severity, impairment, quality of life, and health care utilization will serve as outcomes. We want to answer the following three research questions:

1. In which psychological factors do patients with SSD/BDD differ compared to control groups?
2. How are psychological factors associated with disorder-relevant outcomes such as symptom severity, functional impairment, health care utilization, and quality of life?
3. Which psychological factors are predictive for the development or maintenance of SSD/BDD?

#### Data synthesis and planned analyses

Data of study characteristics and methods employed will be descriptively summarized in Tables 3, 4.

**Table 3:** Information on included studies with fictional examples.

Study authors (year)	Type of study	Study characteristics	Diagnosis and assessment	Psychological variables (measurement tool)	Outcome(s)	Effect size
Example et al. (2015)	RCT on DBT therapy in patients with SSD	200 patients with SSD undergoing DBT vs. control (waiting list)	SSD: Cut-off scores (PHQ-15 ⩾ 15 and SSD-12 ⩾ 15)	Depression (PHQ-9), Somatosensory amplification (SSAS)	Primary: Diagnosis present at follow-up (cut-off scores), Secondary: symptom severity (NRS)	PHQ-9: d = xx, SSAS: d = yy; NRS: d = zz
Illustration (2016)	Cohort study on the development of BDD and the association with somatosensory amplification/traumatic childhood experiences	Cohort of 342 patients (BDD vs. no BDD)	BDD: clinical judgment	Somatosensory amplification (SSAS), Traumatic experience (ACE)	Somatosensory amplification score in BDD sample	*r* = aa (SSAS and ACE), *d* = bb

DBT, Dialectic Behavioral Therapy; SSD, Somatic Symptom Disorder; BDD, Bodily Distress Disorder; PHQ-9, Patient Health Questionnaire-9; PHQ-15, Patient Health Questionnaire-15; SSAS, Somatosensory Amplification Scale; SF-12, Short Form 12 Health Survey; NRS, numeric rating scale; ACE, Adverse Childhood Experiences Questionnaire.

**Table 4:** Psychological variables and effect sizes of associations on disorder-relevant factors.

Category	Sub-category	Variable	Studies providing data	Outcome(s)	Effect sizes of variable and outcome association
Psychopathology	Depression	Depressive symptoms	Example et al. (2015)	SSD diagnosis	d = xx
Cognitive	Attentional processes	Somatosensory amplification	Example et al. (2015), Illustration (2016)	SSD diagnosis, SSAS score in BDD sample	d = yy, d = bb

SSD, somatic symptom disorder; BDD, bodily distress disorder.

While Table 3 includes an overview of all included studies, Table 4 lists the empirical evidence for each psychological factor identified throughout the review process.

If at least three studies are available for the same outcome and psychological variable, meta-analyses will be performed. If meta-analysis is not appropriate, we will report outcomes narratively. For conducting meta-analyses we will use the statistical software R (version 4.1.1) (55). Due to anticipated heterogeneity of diagnoses, populations and outcome measures included in our data, we do not expect to conceive collected effect sizes to represent a single population effect size. We will thus conduct random-effects analyses for each outcome. We will compute weights using the inverse-variance method. Between-study variance (τ2) will be estimated the method of restricted maximum likelihood (56, 57). We will report a summary effect and its corresponding 95% confidence interval using the Knapp-Hartung method and its 95% prediction interval (58, 59).

#### Study registration

The framework for this review project has been registered with PROSPERO [registration number CRD42022302014 (60)]. Both lists comprising the search terms for the psychological variables and the terms and synonyms for PSS and related syndromes and disorders are available on the Open Science Framework OSF (49).

## Discussion

With this publication, we provide a framework to systematically review the current evidence on psychological associates and risk factors in persistent somatic symptoms and related syndromes and disorders. We further illustrate the use of this framework by presenting the protocol for a first systematic review on the evidence of the associates and risk factors of psychological factors in SSD and BDD, the diagnostic classifications currently in use in DSM-5 and ICD-11.

Thereby, we hope to provide sound evidence for the ongoing debate on the relevance of psychological features for the diagnosis of PSS and related syndromes and disorders. This debate has been going on since the introduction of the DSM-5 SSD criteria, with critics arguing that psychological features are neither necessary nor sufficient to make valid diagnoses in patients with PSS and related syndromes and disorders (61, 62). However, there is evidence that psychological features predict outcomes relevant to PSS, such as stability of diagnosis, health care utilization, or quality of life (24, 27). As such, a recently proposed alternative classification for PSS, i.e., “functional somatic disorders,” does not rely on psychological features, but proposes their additional specification, thereby giving priority to features that are prognostic in terms of severity/duration or guiding treatment (61).

In this sense, the results to be derived from our framework will provide a starting point for an improved mechanistic understanding and refinement of future diagnostic conceptualizations for PSS and related syndromes and disorders. The identification of relevant psychological factors holds the great chance that these are modifiable and, therefore, a promising gateway to improve the currently only moderately effective treatment for PSS and related syndromes and disorders (37, 47, 63). Targeted interventions for prevention, early identification and treatment of PSS and related syndromes and disorders can only be developed once psychological risk factors and mechanisms are better understood.

A major strength of our framework is that it provides a valid and comprehensive foundation that has been derived through a sound systematic approach. It will enable researchers to flexibly carry out systematic reviews with broad yet also specific aims (i.e., selection of only a sub-group of psychological variables and/or diagnoses). As major limitations, we acknowledge that both lists may not be complete, are mostly limited to current evidence from Western countries, and need to be updated regularly based on new evidence. We thus advise interested researcher to always visit the ORF for the latest versions of both PSY-PSS lists (49).

Also, the classification of the psychological constructs into (sub-) categories may be debatable, as it is based on an inductive process of expert opinion. However, we believe our framework could be a useful starting point for consecutive reviews that will improve the current understanding and knowledge in diagnosing and treating PSS and related syndromes and disorders. Thus, we explicitly invite research groups from different medical and psychological specialties to use and further develop the PSY-PSS framework with respect to their individual research questions. The PSY-PSS framework can further be extended to investigate evidence on psychological factors in symptom-based medical diseases such as inflammatory bowel disease or rheumatologic conditions.

The ongoing debate on DSM-5 somatic symptom disorder and the upcoming implementation of ICD-11 in health-care systems worldwide requires additional research. ICD-11 is a classification system which is designed to be open to change and adaptations, and thus reliant on constantly updated empirical evidence. In the end, not only research, but also especially clinical care will benefit from a better knowledge of what to include in the so-called “psycho-bundle” of PSS and related syndromes and disorders.

## Author contributions

AT, MS-M, and PH were involved in the concept and design of the study. AT and PH conducted the preliminary search. Feedback on all variables was provided by BL and MK. Further internal discussion involved AT, MS-M, PH, and AS. PH wrote the draft of this manuscript. AT, MS-M, BL, MK, and AS provided valuable revisions. All authors contributed to the article and approved the submitted version.

## Funding

We acknowledge financial support from the Open Access Publication Fund of UKE-Universitätsklinikum Hamburg-Eppendorf- and DFG – German Research Foundation.

## Conflict of interest

The authors declare that the research was conducted in the absence of any commercial or financial relationships that could be construed as a potential conflict of interest.

## Publisher’s note

All claims expressed in this article are solely those of the authors and do not necessarily represent those of their affiliated organizations, or those of the publisher, the editors and the reviewers. Any product that may be evaluated in this article, or claim that may be made by its manufacturer, is not guaranteed or endorsed by the publisher.
